# Consumers’ Preferences for Purchasing mHealth Apps: Discrete Choice Experiment

**DOI:** 10.2196/25908

**Published:** 2023-09-13

**Authors:** Zhenzhen Xie, Calvin Kalun Or

**Affiliations:** 1 Department of Industrial and Manufacturing Systems Engineering, University of Hong Kong, Hong Kong, China (Hong Kong)

**Keywords:** consumer preferences, discrete choice experiment, DCE, mobile health, mHealth, digital health

## Abstract

**Background:**

There is growing interest in mobile health apps; however, not all of them have been successful. The most common issue has been users’ nonadoption or abandonment of health apps because the app designs do not meet their preferences. Therefore, to facilitate design-preference fit, understanding consumers’ preferences for health apps is necessary, which can be accomplished by using a discrete choice experiment.

**Objective:**

This study aims to examine consumer preferences for health apps and how these preferences differ across individuals with different sociodemographic characteristics and health app usage and purchase experiences.

**Methods:**

A cross-sectional discrete choice experiment questionnaire survey was conducted with 593 adults living in Hong Kong. A total of 7 health app attributes that might affect consumers’ preferences for health apps were examined, including usefulness, ease of use, security and privacy, health care professionals’ attitudes, smartphone storage consumption, mobile data consumption, and cost. Mixed-effect logit regressions were used to examine how these attributes affected consumer preferences for health apps. Fixed effects (coefficient β) of the attributes and random effects of individual differences were modeled. Subgroup analyses of consumer preferences by sex, age, household income, education level, and health app usage and purchase experiences were conducted.

**Results:**

Cost was the attribute that had the greatest effect on consumers’ choice of health apps (compared to HK $10 [US $1.27]—HK $50 [US $6.37]: β=−1.064; *P*<.001; HK $100 [US $12.75]: β=−2.053; *P*<.001), followed by security and privacy (compared to *no security insurance*—*some security policies*: β=.782; *P*<.001; *complete security system*: β=1.164; *P*<.001) and usefulness (compared to *slightly useful*—*moderately useful*: β=.234; *P*<.001; *very useful*: β=.979; *P*=.007), mobile data consumption (compared to *data-consuming*—*a bit data-consuming*: β=.647; *P*<.001; *data-saving*: β=.815; *P*<.001), smartphone storage consumption (compared to >100 MB—around 38 MB: β=.334; *P*<.001; <10 MB: β=.511; *P*<.001), and attitudes of health care professionals (compared to *neutral*—*moderately supportive*: β=.301; *P*<.001; *very supportive*: β=.324; *P*<.001). In terms of ease of use, consumers preferred health apps that were moderately easy to use (compared to *not easy to use*—*moderately easy to use*: β=.761; *P*<.001; *very easy to use*: β=.690; *P*<.001). Our results also showed that consumers with different sociodemographic characteristics and different usage and purchase experiences with health apps differed in their preferences for health apps.

**Conclusions:**

It is recommended that future health apps keep their mobile data and phone storage consumption low, include a complete security system to protect personal health information, provide useful content and features, adopt user-friendly interfaces, and involve health care professionals. In addition, health app developers should identify the characteristics of their intended users and design and develop health apps to fit the preferences of the intended users.

## Introduction

With the rising prevalence of mobile health (mHealth) apps [1-3] and the accumulating evidence of their effectiveness in improving health outcomes [4,5], interest in developing health apps has continued to grow. mHealth apps have been applied to support health care activities, such as disease detection, patient monitoring, health data collection, remote diagnosis, and disease management [6-10]. However, the implementation of health apps is not always easy, and several attempts have not achieved the desired results [11,12]. The most common issue has been the nonadoption or abandonment of health apps by consumers [13], indicating a gap between the health app and consumers’ preferences and highlighting the need to understand which characteristics of health apps affect consumer preferences for health apps [14,15].

To obtain such knowledge, we can use a discrete choice experiment (DCE), which is a research technique that elicits consumers’ stated preferences for products or services and assesses the contribution of various characteristics (ie, the attributes of the products or services) to those preferences [16-18]. In a DCE, researchers predetermine the attributes that may potentially affect consumer choices and the levels for each attribute of the target product or service. Researchers then create hypothetical alternatives of the product or service by combining the different levels of those attributes. Finally, researchers ask participants about which alternatives they would be willing to purchase; this information reflects their preferences for the alternatives. The participants’ responses can be used to derive information regarding how consumer preferences are affected by each of the attributes [19]. With such information, product and service designers and developers would become aware of the attributes that consumers care about, allowing them to focus more on the influential attributes for better product and service designs.

DCEs have been applied to understand consumer preferences for different health technologies, including telehealth systems and appointment reminder systems [20-22]. These studies have demonstrated that DCEs can generate useful information from consumers’ perspectives for guiding health technology design. However, among the research related to health apps, the use of DCEs to examine consumer preferences remains scarce, and little is known about which attributes of health apps should be prioritized during the development of new health apps. Therefore, in this study, we aimed to use a DCE to examine consumer preferences for health apps. In addition, as consumers with different sociodemographic characteristics and health app usage levels may have different preferences toward health apps, we also aimed to examine consumer preferences for health apps across individuals with different sociodemographic characteristics, health app usage experiences, and health app purchase experiences.

## Methods

### Questionnaire Development

A questionnaire was developed to collect data on participants’ sociodemographics (sex, age, district of residence, household size, household monthly income, and education level), usage of health apps, previous purchases of health apps, and preferences for each attribute of health apps. Usage of health apps was assessed by asking participants to indicate whether they had health apps installed on their smartphone. Previous purchases of health apps were measured by asking participants whether they had paid for health apps before. Consumer preferences were assessed by using a set of DCE questions, which were developed as described below.

**Figure 1.**
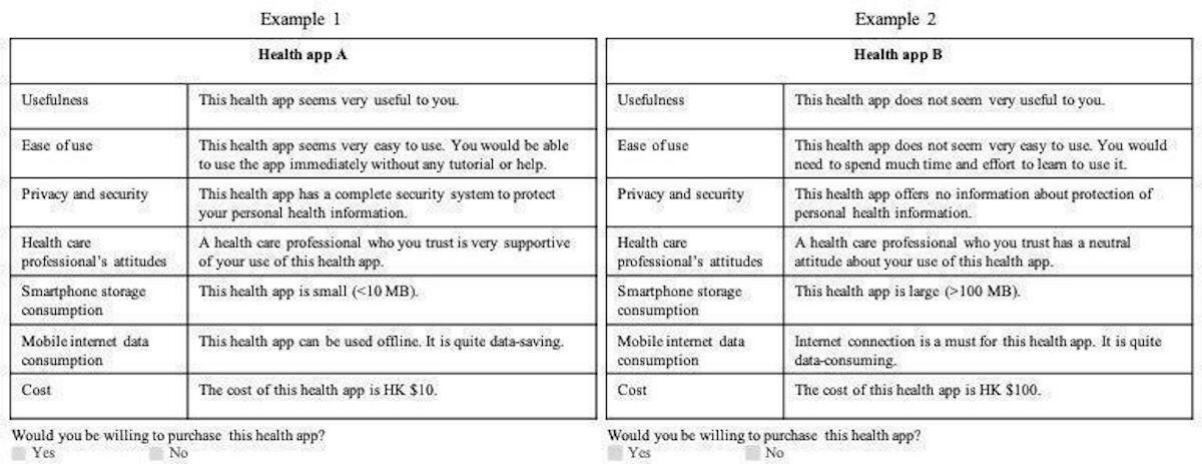
Two examples of the DCE question designed to familiarize the participants with the format of the questions and test whether participants understood the DCE questions. A currency exchange rate of HK $1=US $0.13 is applicable. DCE: discrete choice experiment.

We carefully reviewed the literature on the factors that affect individuals’ decision to use health apps [23-38] in order to identify health app attributes that may contribute to consumers’ preferences for health apps. We then consolidated these attributes and assigned 3 levels to each one (Table 1). Based on these attributes and levels, 18 hypothetical health apps were formed, using an orthogonal factorial design. For each hypothetical health app, we created a question that asked whether participants would be willing to purchase the app. Prior to answering these questions, participants were asked to answer 2 example questions (Figure 1) to familiarize themselves with the format of the questions. These example questions were also used to test the validity of participants’ answers; as health app A in example 1 is superior to health app B in example 2 for every attribute, participants who refused to purchase health app A but chose to purchase health app B may have not fully understood the questions, and their responses were excluded from data analysis.

**Table 1.** Attributes and levels in the discrete choice experiment.

Attributes and levels		Descriptions
**Usefulness**		
	Slightly useful	This health app seems slightly useful to you.
	Moderately useful	This health app seems moderately useful to you.
	Very useful	This health app seems very useful to you.
**Ease of use**		
	Not easy to use	This health app does not seem very easy to use. You would need to spend much time and effort to learn to use it.
	Moderately easy to use	This health app seems moderately easy to use. You could learn to use it quickly.
	Very easy to use	This health app seems very easy to use. You would be able to use the app immediately without any tutorial or help.
**Security and privacy**		
	No security assurance	This health app offers no information about protection of personal health information.
	Some security assurance	This health app provides some information about security policies related to personal health information.
	Complete security system	This health app has a complete security system to protect your personal health information.
**Health care professional’s attitude**		
	Neutral attitude	A health care professional who you trust has a neutral attitude about your use of this health app.
	Moderately supportive	A health care professional who you trust is moderately supportive of your use of this health app.
	Very supportive	A health care professional who you trust is very supportive of your use of this health app.
**Smartphone storage consumption (MB)**		
	>100	This health app is large (>100 MB).
	Around 38	This health app is medium (around 38 MB).
	<10	This health app is small (<10 MB).
**Mobile internet data consumption**		
	Quite data-consuming	Internet connection is a must for this health app. It is quite data-consuming.
	A bit data-consuming	Some functions of this health app require an internet connection. It is a bit data-consuming.
	Quite data-saving	This health app can be used offline. It is quite data-saving.
**Cost (HK $^a^)**		
	10	The cost of this health app is HK $10.
	50	The cost of this health app is HK $50.
	100	The cost of this health app is HK $100.

a A currency exchange rate of HK $1=US $0.13 is applicable.

### Data Collection

The questionnaire was distributed to adults (aged ≥18 y) residing in Hong Kong, and participant recruitment was stratified by age, sex, and district of residence according to the population distribution [39]. Trained research assistants approached potential participants in public places, explaining this study and determining their eligibility. Individuals were eligible if they knew what health apps were and were able to understand and answer the questionnaire. Eligible participants were asked to provide written informed consent and then answer the questionnaire. To ensure that all participants had the same understanding of what health apps were, research assistants provided the following definition of *health apps* to each participant: health apps are software programs installed on smartphones that aim to assist health management. Research assistants also provided examples of health apps, including those that track health indicators, send health reminders, present health information, promote self-management behaviors and healthy lifestyles, and provide remote monitoring and diagnosis. Upon finishing the survey, each participant was given a supermarket coupon for HK $50 (US $6.37).

### Data Analysis

Descriptive statistics were used to reflect the study sample’s sociodemographic characteristics and their health app usage and purchase experiences. A mixed-effect logit model was used to examine consumer preferences for health apps by examining how each attribute of a health app affects whether participants would choose to purchase the health app. Fixed effects of the seven attributes and random effects of individual differences were modeled. Coefficients of the fixed effects (β), their SEs, and corresponding *P* values were reported. In addition, subgroup analyses of consumer preferences by sex, age, household income, education level, and health app usage and purchase experiences were conducted. All statistical analyses were performed by using R 4.0.2 software (R Foundation for Statistical Computing).

### Ethical Considerations

This study received ethical approval from the Human Research Ethics Committee of the University of Hong Kong (approval number: EA1810020).

## Results

### Sample Characteristics

Among the 600 individuals who completed the DCE, 7 did not fully understand the discrete choice question, judging from their responses to the two example questions of the DCE. Therefore, the study sample consisted of 593 adults; 47.2% (280/593) of them had health apps installed on smartphones, and 10.5% (62/593) had paid for a health app before. Sociodemographic characteristics of the study sample are presented in Table 2.

**Table 2.** Sociodemographic characteristics of the study sample (N=593).

Characteristics		Values
Age (years), mean (SD)		45.96 (15.85)
**Sex, n (%)**		
	Female	318 (53.6)
	Male	275 (46.4)
**Monthly household income (HK $^a^), n (%)**		
	<10,000	47 (7.9)
	10,000-19,999	122 (20.6)
	20,000-29,999	113 (19.1)
	30,000-39,999	68 (11.5)
	40,000-49,999	81 (13.7)
	50,000-79,999	86 (14.5)
	≥80,000	76 (12.8)
**Education level, n (%)**		
	Primary school or below	64 (10.8)
	Some secondary school or completed secondary school	222 (37.4)
	Postsecondary degree (diploma, bachelor, master, or doctoral degree)	307 (51.8)

a A currency exchange rate of HK $1=US $0.13 is applicable.

### Consumer Preferences for Health Apps

Table 3 presents the results of the mixed logit regression that examined how each attribute of a health app affects whether participants would be willing to purchase the health app.

**Table 3.** Mixed logit regression that examined how each attribute of a health app affects whether participants would be willing to purchase the health app.

Attributes and levels		β (SE)	*P* value
**Usefulness (reference level: slightly useful)**			
	Moderately useful	.234 (0.086)	<.001
	Very useful	.979 (0.081)	.007
**Ease of use (reference level: not easy to use)**			
	Moderately easy to use	.761 (0.090)	<.001
	Very easy to use	.690 (0.080)	<.001
**Security and privacy (reference level: no security assurance)**			
	Some security policies	.782 (0.082)	<.001
	Complete security system	1.164 (0.084)	<.001
**Health care professional’s attitude (reference level: neutral)**			
	Moderately supportive	.301 (0.082)	<.001
	Very supportive	.324 (0.081)	<.001
**Smartphone storage consumption (MB; reference level: >100)**			
	Around 38	.334 (0.082)	<.001
	<10	.511 (0.081)	<.001
**Mobile data consumption (reference level: data-consuming)**			
	A bit data-consuming	.647 (0.081)	<.001
	Data-saving	.815 (0.081)	<.001
**Cost (HK $^a^; reference level: 10)**			
	50	−1.064 (0.075)	<.001
	100	−2.053 (0.086)	<.001

a A currency exchange rate of HK $1=US $0.13 is applicable.

### Subgroup Analysis of Consumer Preferences for Health Apps

Tables 4-6 present results of the mixed logit regressions by subgroups of sex, age, monthly household income, education level, whether participants have health apps installed on their smartphone, and whether participants have paid for health apps before.

**Table 4.** Mixed logit regressions that examined how each attribute of a health app affects whether participants would be willing to purchase the health app by subgroups of sex and age.

Attributes and levels		Sex		Age	
		Male, β (SE)	Female, β (SE)	Younger (≤45 years), β (SE)	Older (>45 years), β (SE)
**Usefulness (reference level: slightly useful)**					
	Moderately useful	.244 (0.114)^a^	.241 (0.131)	.416 (0.121)^a^	.018 (0.125)
	Very useful	.916 (0.109)^b^	1.079 (0.121)^a^	1.350 (0.113)^a^	.546 (0.118)^a^
**Ease of use (reference level: not easy to use)**					
	Moderately easy to use	.749 (0.121)^a^	.779 (0.132)^a^	.706 (0.122)^a^	.851 (0.133)^a^
	Very easy to use	.682 (0.107)^a^	.681 (0.120)^a^	.645 (0.108)^a^	.763 (0.119)^a^
**Security and privacy (reference level: no security assurance)**					
	Some security policies	.955 (0.110)^a^	.567 (0.121)^a^	1.022 (0.113)^a^	.525 (0.120)^a^
	Complete security system	1.260 (0.114)^a^	1.043 (0.121)^a^	1.392 (0.116)^a^	.914 (0.121)^a^
**Health care professional’s attitude (reference level: neutral)**					
	Moderately supportive	.316 (0.110)^c^	.273 (0.121)^b^	.488 (0.113)^a^	.114 (0.120)
	Very supportive	.334 (0.109)^c^	.301 (0.120)^b^	.525 (0.113)^a^	.117 (0.117)
**Smartphone storage consumption (MB; reference level: >100)**					
	Around 38	.401 (0.109)^a^	.260 (0.122)^b^	.335 (0.112)^c^	.382 (0.120)^c^
	<10	.473 (0.110)^a^	.574 (0.118)^a^	.392 (0.111)^a^	.680 (0.119)^a^
**Mobile data consumption (reference level: data-consuming)**					
	A bit data-consuming	.603 (0.108)^a^	.743 (0.122)^a^	.646 (0.110)^a^	.637 (0.119)^a^
	Data-saving	.751 (0.108)^a^	.915 (0.123)^a^	.859 (0.109)^a^	.760 (0.121)^a^
**Cost (HK $** ^**d**^ **; reference level: 10)**					
	50	−.871 (0.103)^a^	−1.251 (0.107)^a^	−1.236 (0.102)^a^	−.882 (0.111)^a^
	100	−1.744 (0.113)^a^	−2.417 (0.133)^a^	−2.181 (0.116)^a^	−1.925 (0.128)^a^

a Significant at the *P*<.001 level.

b Significant at the *P*<.05 level.

c Significant at the *P*<.01 level.

d A currency exchange rate of HK $1=US $0.13 is applicable.

**Table 5.** Mixed logit regressions that examined how each attribute of a health app affects whether participants would be willing to purchase the health app by subgroups of monthly household income and education level.

Attributes and levels		Monthly household income		Education level	
		Lower (<HK $30,000^a^), β (SE)	Higher (≥HK $30,000), β (SE)	Lower (completed secondary school), β (SE)	Higher (postsecondary degree), β (SE)
**Usefulness (reference level: slightly useful)**					
	Moderately useful	.239 (0.130)	.225 (0.114)^b^	.034 (0.124)	.408 (0.121)^c^
	Very useful	.734 (0.124)^c^	1.156 (0.106)^c^	.497 (0.118)^c^	1.388 (0.114)^c^
**Ease of use (reference level: not easy to use)**					
	Moderately easy to use	.687 (0.137)^c^	.818 (0.117)^c^	.828 (0.133)^c^	.704 (0.122)^c^
	Very easy to use	.662 (0.121)^c^	.713 (0.105)^c^	.707 (0.118)^c^	.689 (0.108)^c^
**Security and privacy (reference level: no security assurance)**					
	Some security policies	.682 (0.123)^c^	.867 (0.108)^c^	.575 (0.119)^c^	.980 (0.113)^c^
	Complete security system	.996 (0.127)^c^	1.293 (0.110)^c^	.925 (0.122)^c^	1.366 (0.116)^c^
**Health care professional’s attitude (reference level: neutral)**					
	Moderately supportive	.272 (0.124)^b^	.326 (0.107)^d^	.231 (0.120)	.394 (0.112)^c^
	Very supportive	.239 (0.123)	.381 (0.106)^c^	.140 (0.117)	.483 (0.113)^c^
**Smartphone storage consumption (MB; reference level: >100)**					
	Around 38	.459 (0.125)^c^	.242 (0.106)^b^	.359 (0.120)^d^	.346 (0.112)^c^
	<10	.667 (0.123)^c^	.390 (0.106)^c^	.682 (0.119)^c^	.387 (0.112)^c^
**Mobile data consumption (reference level: data-consuming)**					
	A bit data-consuming	.509 (0.123)^c^	.747 (0.106)^c^	.408 (0.118)^c^	.843 (0.112)^c^
	Data-saving	.744 (0.123)^c^	.866 (0.107)^c^	.548 (0.119)^c^	1.045 (0.111)^c^
**Cost (HK $; reference level: 10)**					
	50	−1.073 (0.115)^c^	−1.066 (0.098)^c^	−1.043 (0.112)^c^	−1.122 (0.102)^c^
	100	−2.173 (0.132)^c^	−1.971 (0.112)^c^	−2.126 (0.129)^c^	−2.035 (0.116)^c^

a A currency exchange rate of HK $1=US $0.13 is applicable.

b Significant at the *P*<.05 level.

c Significant at the *P*<.001 level.

d Significant at the *P*<.01 level.

**Table 6.** Mixed logit regressions that examined how each attribute of a health app affects whether participants would be willing to purchase the health app by subgroups of health app usage and purchase experiences.

Attributes and levels		Has health apps installed on smartphone		Has paid for health apps before	
		No, β (SE)	Yes, β (SE)	No, β (SE)	Yes, β (SE)
**Usefulness (reference level: slightly useful)**					
	Moderately useful	.041 (0.134)	.384 (0.112)^a^	.246 (0.098)^b^	.270 (0.198)
	Very useful	.778 (0.123)^a^	1.131 (0.107)^a^	.973 (0.092)^a^	1.226 (0.200)^a^
**Ease of use (reference level: not easy to use)**					
	Moderately easy to use	.562 (0.138)^a^	.920 (0.117)^a^	.619 (0.100)^a^	1.354 (0.218)^a^
	Very easy to use	.637 (0.122)^a^	.737 (0.104)^a^	.628 (0.088)^a^	.991 (0.195)^a^
**Security and privacy (reference level: no security assurance)**					
	Some security policies	.767 (0.126)^a^	.800 (0.107)^a^	.829 (0.091)^a^	.559 (0.194)^c^
	Complete security system	1.065 (0.128)^a^	1.252 (0.109)^a^	1.179 (0.094)^a^	.999 (0.200)^a^
**Health care professional’s attitude (reference level: neutral)**					
	Moderately supportive	.192 (0.125)	.379 (0.108)^a^	.317 (0.092)^a^	.311 (0.197)
	Very supportive	.216 (0.122)	.412 (0.106)^a^	.376 (0.091)^a^	.185 (0.193)
**Smartphone storage consumption (MB reference level: >100)**					
	Around 38	.273 (0.124)^b^	.381 (0.107)^a^	.389 (0.091)^a^	.101 (0.195)
	<10	.519 (0.123)^a^	.503 (0.106)^a^	.568 (0.090)^a^	.217 (0.197)
**Mobile data consumption (reference level: data-consuming)**					
	A bit data-consuming	.739 (0.124)^a^	.567 (0.105)^a^	.740 (0.090)^a^	.251 (0.191)
	Data-saving	.744 (0.126)^a^	.871 (0.105)^a^	.852 (0.091)^a^	.727 (0.196)^a^
**Cost (HK $** ^**d**^ **; reference level: 10)**					
	50	−.920 (0.111)^a^	−1.183 (0.101)^a^	−1.096 (0.081)^a^	−.797 (0.198)^a^
	100	−2.062 (0.134)^a^	−2.050 (0.111)^a^	−2.223 (0.097)^a^	−1.331 (0.198)^a^

a Significant at the *P*<.001 level.

b Significant at the *P*<.05 level.

c Significant at the *P*<.01 level.

d A currency exchange rate of HK $1=US $0.13 is applicable.

## Discussion

### Principal Findings

Our results showed that cost was the attribute that had the greatest influence on consumers’ preferences for health apps, followed by security and privacy and then by usefulness. Consumers also preferred health apps that used less mobile data and took up less smartphone storage, as well as health apps that health care professionals held positive attitudes toward. In terms of ease of use, consumers preferred health apps that were moderately easy to use over those that were very easy to use, and both were preferred over those that were not easy to use. Our results also showed that consumers with different sociodemographic characteristics and different usage and purchase experiences with health apps differed in their preferences for health apps. Understanding consumer preferences in these subgroups could be informative for developing health apps that target consumers in these subgroups.

Consumers preferred health apps that cost less money and consumed less mobile data and smartphone storage. This finding is consistent with previous research that found that cost was the greatest concern for using health apps and that many individuals would not pay anything for a health app [25]. This is also consistent with findings from previous research in which the consumption of resources was a major barrier to using health apps [27,34,35]. It is therefore suggested that health app developers optimize the app size by identifying and removing unnecessary files and codes, reducing the size of images and videos, and providing on-demand downloads for less frequently used resources. Health app developers are also suggested to optimize the data usage of the app by reducing automatic data loading, paginating large volumes of data, and using small-sized images for previews. These approaches could help keep the use of health apps at low costs, which is crucial for scaling up and spreading the use of health apps.

Consumers also strongly preferred health apps with a complete security system to protect their personal health information. This may be explained by findings from previous research in which the concerns for the security of personal health information collected by health apps led to a lack of trust in health apps and constituted a major barrier for the use of health apps [3,25,40]. The fact that many health apps have yet to deploy appropriate techniques for protecting the security and privacy of users [41-43] could constitute a major issue that hampers consumers’ adoption of health apps. It is suggested that health app developers should take measures (eg, data encryption, data integrality, and freshness protection) to upgrade the security level of users’ personal health information stored in or transmitted through health apps [44]. It is also suggested that mobile app platforms and policy makers launch guidelines, implement policies, and impose regulations to ensure that all health apps can properly protect the privacy of their users [45,46].

We also found that the usefulness and ease of use of health apps influenced consumers’ preferences for them. These two attributes have long been considered to be the major reasons why people accept or choose to use health technology in the literature [28,47-51]. Specifically, we found that consumers would always prefer a health app that was more useful, underscoring the importance of understanding users’ actual needs and how the health app can be helpful in fulfilling these needs [52]. As for ease of use, consumers most preferred health apps that were moderately easy to use, indicating that consumers preferred health apps that were user-friendly but did not seem too simple. This is probably because consumers perceived health apps that were too easy to use as being too simple to be worthy of purchase. It is therefore suggested that human factors design principles are followed in the design and development of health apps to ensure that they are user-friendly [53-58] and that the useful content, features, and functionalities of the health app are highlighted when promoting the app to its intended users.

Consumers also preferred health apps that health care professionals had positive attitudes toward, most likely because they believed that health care professionals have more knowledge about health management and trusted health care professionals’ judgments about health-related products. This is consistent with findings from previous research in which individuals were more willing to use health apps if they were recommended by health care professionals [59,60]. It is thus suggested that the involvement of health care professionals can be effective in the promotion of health apps.

### Limitations

This study has limitations. First, the DCE used hypothetical scenarios to elicit consumers’ stated preferences, without requiring real economic commitments (ie, actual purchases). As shown in previous research that found that hypothetical products were usually valued higher than actual products, the responses obtained by using a DCE may be affected by hypothetical bias and differ from consumers’ behavior in real life [61-64]. Second, we adopted an orthogonal factorial design in the DCE, which enabled us to examine the main effects of each attribute but ignored the interaction effects between these attributes [19]. Future work can be conducted to examine how the interactions between attributes affect consumers’ preferences for health apps. Third, the choice task used in this study presented participants with only 1 hypothetical health app and asked them to choose if they would like to purchase it. We were thus unable to observe how consumers compared and chose among multiple health apps and assess how they traded off between different attributes of health apps.

### Conclusions

Health apps are preferable when they cost less, consume less storage and mobile data, can protect the security and privacy of personal health data, are useful and easy to use, and are recommended by health care professionals. Therefore, it is recommended that future health apps keep their cost, mobile data consumption, and phone storage consumption low; include a complete security system to protect personal health information; provide useful content and features; adopt user-friendly interfaces; and involve health care professionals. In addition, health app developers should identify the characteristics of their intended users and design and develop health apps to fit the preferences of the intended users.

## Abbreviations

DCE: discrete choice experiment
mHealth: mobile health
